# OEA alleviates apoptosis in diabetic rats with myocardial ischemia/reperfusion injury by regulating the PI3K/Akt signaling pathway through activation of TRPV1

**DOI:** 10.3389/fphar.2022.964475

**Published:** 2022-11-14

**Authors:** Enhui Yao, Lili Luo, Chenxi Lin, Jing Wen, Yanglongfei Li, Tong Ren, Yujie Chen, Jinhua Huang, Xin Jin

**Affiliations:** ^1^ Department of Cardiology, Fujian Medical University Union Hospital, Fujian Institute of Coronary Artery Disease, Fujian Heart Medical Center, Fuzhou, China; ^2^ Xiamen Key Laboratory of Chiral Drugs, School of Medicine, Xiamen University, Xiamen, China; ^3^ Department of Pediatrics, Zhongshan Hospital of Xiamen University, School of Medicine, Xiamen University, Xiamen, China

**Keywords:** oleoylethanolamide, myocardial ischemia-reperfusion injury, diabetic rats, apoptosis, TRPV1

## Abstract

Reperfusion therapy after myocardial infarction may lead to myocardial injury, which can be complicated and exacerbated by diabetes. The existing therapeutic methods for myocardial ischemia-reperfusion injury (MIRI) in diabetic patients are not ideal. Oleoylethanolamide (OEA) has been found to have protective effects on diabetes and acute cerebral ischemia. This study aimed to determine whether OEA can alleviate MIRI in diabetic rats, and to explore the underlying mechanism. The model of diabetic rats with MIRI was established by blocking the left coronary artery for 30 min, followed by restoring blood flow stability for 120 min. The myocardial enzyme spectrum, area of MIRI, and expression levels of apoptosis-related proteins were detected. The results showed that OEA pretreatment could reduce myocardial infarction area, protect myocardial tissue structure, and reduce myocardial cell apoptosis in diabetic rats with MIRI. Meanwhile, the levels of creatine kinase (CK)-MB (CK-MB), lactate dehydrogenase (LDH), and malondialdehyde (MDA) were reduced, while superoxide dismutase (SOD) level was elevated. H9C2 cells were treated with high glucose and oxygen-glucose deprivation/reperfusion (OGD/R) to establish an *in vitro* model. Capsazepine (CPZ), an antagonist of transient receptor potential vanilloid subtype 1 (TRPV1), and LY294002, an inhibitor of PI3K, were used to treat H9C2 cells *in vitro*. Apoptosis level and the expression levels of apoptosis-related proteins were measured. It was found that OEA activated TRPV1 and the PI3K/Akt signaling pathway, downregulated the expression levels of apoptosis-related proteins (Bcl-2 and cleaved caspase-3), and ameliorated the apoptosis of H9C2 cells treated with high glucose and OGD/R. This study clarified that OEA, as a TRPV1 agonist, could reduce myocardial cell apoptosis by activating the PI3K/Akt signaling pathway in diabetic rats with MIRI. The findings may provide a theoretical basis for administration of OEA as a potential therapeutic agent into diabetic patients with MIRI.

## Introduction

Ischemic heart disease (IHD) is a leading cause of death worldwide. In 2017, according to the statistics, 1,612 per 1,00,000 people suffer from IHD in China (Zhao et al., 2019). The rapid recovery of blood flow in myocardial ischemia area is an effective treatment, while reperfusion may also induce myocardial injury, which is known as ischemia-reperfusion (I/R) injury (Mokhtari et al., 2020; Naseroleslami et al., 2020). Myocardial I/R injury (MIRI) can lead to the poor prognosis of patients with heart failure and myocardial inflammation, and triggering of ventricular arrhythmias may result in myocardial infarction (Majidi et al., 2015). Clinical data showed that diabetic patients are more prone to MIRI, and the mortality rate after acute myocardial infarction or coronary artery bypass grafting is twice that of non-diabetic patients (Miki et al., 2012; Godoy and Farkouh, 2019). The mechanisms underlying MIRI in diabetic patients are complex, and studies have shown that the excessive production of free radicals and proinflammatory cytokines, the increase of apoptosis, and dysregulation of autophagy are all involved in the development of MIRI (Bayrami et al., 2018; Penna et al., 2020). It has been found that reperfusion provides essential oxygen and glucose for cell survival, as well as energy for apoptosis (Eefting et al., 2004), which accelerates cell apoptosis (Lejay et al., 2016), and leads to the myocardial structural damage and the reduced cardiac function (Brady et al., 2006).

Apoptosis is an energy-demanding process that occurs through chromatin accumulation, DNA fragmentation, and apoptotic body formation, without causing damage to membrane stability and inflammatory responses (Hausenloy and Yellon, 2004). Apoptosis is involved in the pathophysiological process of MIRI in healthy or diabetic conditions, and it is an important cause of diabetic cardiomyopathy (Cai et al., 2002; Yang et al., 2018). Studies have shown that MIRI induces apoptosis in a variety of ways, including elevated Bax level, reduced Bcl-2 level, and activation of the PI3K/Akt pathway (Lakota, 2016). Prevention of myocardial apoptosis has attracted more and more attention as a new target to protect myocardium from MIRI (Nakamura et al., 2000).

Oleoylethanolamide (OEA) is a bioactive endogenous monounsaturated lipid molecule synthesized by oleic acid and phosphatidylethanolamine *via* N-acyltransferase (NAT) in cell membranes (Bowen et al., 2017). OEA is considered to exert its biological activity mainly through the activation of several receptors, including the transient receptor potential vanilloid subtype 1 (TRPV1), peroxisome proliferator-activated receptor-α (PPARα), G protein-coupled receptor 119 (GPR119), etc. (Appendino et al., 2006; Hind et al., 2015). Studies have revealed that TRPV1 reduces the infarct volume and improves motor coordination and neurobehavioral scores in rats with cerebral I/R injury (Huang et al., 2017; Hurtado-Zavala et al., 2017). After I/R injury, activated TRPV1 triggers Ca^2+^ influx, causing a significant improvement in neuronal apoptosis. Meanwhile, Jiang et al. demonstrated that TRPV1^−/−^ mice had significantly increased myocardial apoptosis levels and enlarged infarct area after the onset of MIRI (Jiang et al., 2018). Recently, it has been reported that TRPV1 is involved in the association between cell proliferation and survival (Buch et al., 2018), and the underlying mechanism is related to the activation of the PI3K/Akt pathway by TRPV1 (Zhai et al., 2020).

It has been shown that OEA possesses multiple biological functions, including scavenging free radicals, anti-inflammation, regulating apoptosis (R Pandey et al., 2009; Gonzalez-Aparicio et al., 2014; Hill et al., 2009; Lueneberg et al., 2011; Mazzola et al., 2009), and improving lipid abnormalities (Yang et al., 2016; Brown et al., 2017; Wu et al., 2019). Our previous studies confirmed that OEA has protective effects on diabetic rats (Ren et al., 2020) and acute cerebral ischemia, which can reduce the volume of cerebellar infarction and relieve cerebral edema (Zhou et al., 2012; Zhou et al., 2017). However, it is essential to clarify whether OEA has protective effects on MIRI in diabetic rats.

Therefore, the present study aimed to indicate whether OEA pretreatment can affect MIRI in diabetic rats, and to explore the underlying mechanism. The results may provide a new insight into the treatment of MIRI in diabetic patients.

## Materials and methods

### Animals

Male Sprague-Dawley rats (age, 6-week-old; body weight, 160–200 g) were purchased from Beijing Vitalihua Co., Ltd. (Beijing, China), and housed in the Animal Centre of Xiamen University (Xiamen, China). The study protocol was approved by the Animal Ethics Experimentation Committee of Xiamen University.

### Establishment of the diabetic rat model

After being habituated to the environment for 1 week prior to experiments, all rats were randomly assigned into diabetic model groups and control group. The diabetic rat model was established with reference to the method presented by Ren et al. (2020). In the diabetic model group, rats were once injected with STZ (Beijing Solarbio Science and Technology Co., Ltd., Beijing, China) (30 mg/kg) intraperitoneally after feeding with high-fat chow (No. XTHF45-1) for 8 weeks. In the control group, rats were fed with normal chow (No. SWS902) for 8 weeks. After STZ injection for 72 h, fasting blood glucose (FBG) test was performed on rats in the diabetic model group, and the FBG level>16.7 mmol/L indicated hyperglycemia.

### I/R and experimental groups

Rats in the I/R model group were anesthetized with 10% chloral hydrate (0.4 mg/kg) by intraperitoneal injection, and then, rats were fixed in the supine position on a heated surgical plate with the tongue secured by a tongue depressor. When stable ventilation was ensured, a tape was used to secure the ventilation catheter to prevent deflection and loosening during the procedure. During the surgery, rats were subjected to electrocardiographic testing using the thoracic lead method. The thorax of rats was opened between the third and fourth ribs of the left sternum and fixed with a chest opener, followed by peeling off the pericardium to complete the heart exposure. The left anterior descending branch of the coronary artery was ligated with a 5-0 or 6-0 non-invasive suture, and the blood flow was cut off by passing a plastic hose of approximately 5 mm in length through the ligature. In the sham-operated group, hearts were threaded without ligature. After 30 min of ischemia, the ligature was cut off for reperfusion for 120 min. The chest cavity was closed and the air was squeezed out by the chest cavity during the suturing process. From 3 days before surgery, rats were given intraperitoneal injection of OEA (Shanghai Macklin Biochemical Co., Ltd., Shanghai, China) at the same time every day. In the previous study, this experiment explored the protective effect of 10–80 mg/kg OEA on diabetic myocardial ischemia-reperfusion injury in rats, and the protective effect of 30–60 mg/kg OEA was more significant. At the same time, in order to preliminarily explore whether OEA has a concentration dependence. Therefore, 30 mg/kg and 60 mg/kg were used as experimental conditions in this study. And rats in the model group were given a corresponding volume of saline intraperitoneally as a control.

Rats were assigned into six groups (*n* = 12 rats in each group): normal sham-operated group (NOR + S), normal myocardial ischemia-reperfusion injury model group (NOR + I/R), diabetic mellitus sham-operated group (DM + S), diabetic mellitus myocardial ischemia-reperfusion injury model group (DM + I/R), diabetic mellitus myocardial ischemia-reperfusion injury with low-dose 30 mg/kg OEA administration group (DM + I/R + OEA30), and the diabetic mellitus myocardial ischemia-reperfusion injury with high-dose 60 mg/kg OEA administration group (DM + I/R + OEA60).

### Biochemical assessment

At the end of reperfusion, the myocardial tissue and serum samples were collected to measure the levels of malondialdehyde (MDA), superoxide dismutase (SOD), glycated hemoglobin (HbA1c), lactate dehydrogenase (LDH), and creatine kinase (CK)-MB (CK-MB). The levels of MDA, SOD, and HbA1c were measured by assay kits (Jiancheng Biotechnology Institute, Nanjing, China) according to the manufacturer’s instructions, and the levels of LDH and CK-MB were measured by automatic biochemical instruments (BS-240VET, Mindray Biochemical Co., Ltd., ShenZhen, China).

### Measurement of myocardial infarct size

The experimental rats were successively subjected to coronary artery ligation for 30 min and reperfusion for 120 min. Rats in each group were randomly selected and injected with 3 ml of 2% Evens-blue staining solution (Sigma-Aldrich, St. Louis, MO, United States) was administered intravenously immediately, and the hearts were removed and placed at −80°C for approximately 5–10 min. Then, heart tissues were sliced up and stained with 0.5% 2,3,5-triphenyl tetrazolium chloride (TTC) solution (Sigma-Aldrich, St. Louis, MO, United States). The blue area indicated normal myocardium and the red and pale areas denoted myocardial injury. The images were analyzed by the ImageJ software.

### Hematoxylin-eosin staining

Hematoxylin-eosin staining kit was purchased from (Jiancheng Biotechnology Institute, Nanjing, China). Select sections with intact tissues without wrinkles, bake them in the oven at 60°C for 2 h and then cool to room temperature. The slides were immersed in xylene (I) for 10 min, xylene (II) for 10 min, 100% ethanol for 5 min, 95% ethanol for 5 min, 75% ethanol for 5 min, 50% ethanol for 5 min, distilled water for 1 min and distilled water for 1 min in turn. The slides were lightly shaken to remove excess water, followed by staining with hematoxylin stain for about 5 min, water rinsing for 3–5 s, followed by ethanol fractionation with 0.2% hydrochloric acid for 3 s and then rapid water washing for 3–5 s. Then eosin staining solution was slurry stained for 30 s followed by 1-2 rinses with color-enhancing solution. Then, the slides were sequentially immersed in 50% ethanol for 2 min, 75% ethanol for 2 min, 95% ethanol for 2 min, 100% ethanol for 2 min, xylene (I) for 5 min and xylene (II) for 5 min. The slides were properly dried in a fume hood and sealed with neutral gum.

### Immunohistochemical staining

After collecting the heart tissue, the tissue was fixed and dehydrated, and then embedded in paraffin for sectioning. The tissue sections were baked, dewaxed, and immersed in citric acid antigen repair solution and kept at a constant temperature of 88°C–90°C for 15 min. Then the slide was cooled to room temperature and the tissue area is circled with a histochemical pen. Referring to the immunohistochemical kit (Shanghai Macklin Biochemical Co., Ltd., Shanghai, China) instructions, tissues were incubated sequentially with endogenous peroxide blockers and nonspecific staining blockers for 10 min at room temperature. Subsequently, the antibody (PI3K-P85, 1:400, 4G3C11, Proteintech) were added to the tissues and incubated overnight at 4°C. The tissue was removed the next day and returned to room temperature, then rinsed three times with PBS buffer.

Tissue sections were incubated with biotin-labeled goat anti-mouse/rabbit polymer at room temperature for 10 min, and then washed three times with PBS buffer for 3 min each time. *Streptomyces* avidin peroxidase was added, incubated at room temperature for 10 min, and washed three times with PBS buffer. Subsequently, the tissue was stained with DAB chromogenic solution to show positive results, and the nucleus was stained with hematoxylin. Finally the sections were sealed with neutral resin.

### Cell culture and establishment of the oxygen-glucose deprivation/reperfusion model

H9C2 cells were cultured in a low-glucose (glucose content, 1 g/L) Dulbecco’s modified Eagle’s medium (DMEM, Gibico, Thermo Fisher Scientific, Waltham, MA, United States), containing 10% fetal bovine serum (FBS, Gibico, Thermo Fisher Scientific, Waltham, MA, United States)), 100 U/mL penicillin, and 100 mg/ml streptomycin (Beijing Solarbio Science and Technology Co., Ltd., Beijing, China), in the presence of 5% CO_2_ and constant humidity at 37°C.

The H9C2 cells in the logarithmic growth phase were uniformly spread according to the corresponding experimental requirements, and then, incubated in an incubator for 24 h. The high-glucose (33.3 mM) medium containing 2% FBS was added to treat the cells for 48 h, resulting in the establishment of an *in vitro* high-glucose model. Afterwards, the culture medium was replaced with a serum-free and sugar-free medium. The plates were placed in a hypoxic chamber. The hypoxic chamber is a special airtight container with inlet and outlet. After putting the cell culture plate into the hypoxic chamber, the valve of outlet port was opened, and the mixed gas containing 5% CO_2_ and 95% N_2_ was filled in the anoxic chamber *via* the inlet port. Immediately close the inlet and outlet valves at the same time, the hypoxic chamber to form an oxygen-free closed environment, and then, the hypoxic chamber is placed in an incubator at 37°C for experiments. After 6 h, the cells were removed from the hypoxic chamber and the medium in the plates was replaced with the medium collected before the OGD. The cells were reoxygenated for 4 h before further testing by the relevant experiments.

Cells at the logarithmic growth phase were seeded into the plates at an appropriate density, and then, incubated overnight in a low-glucose medium supplemented with 10% FBS. According to different treatment conditions, H9C2 cells were assigned into high-glucose treatment group (HG group), high glucose and oxygen-glucose deprivation/reperfusion treatment group (HG + OGD/R group), high glucose and oxygen-glucose deprivation/reperfusion and OEA treatment group (HG + OGD/R + OEA group), high glucose and oxygen-glucose deprivation/reperfusion and LY294002 treatment group (HG + OGD/R + LY294002 group), high glucose and oxygen-glucose deprivation/reperfusion and LY294002 and OEA treatment group (HG + OGD/R + LY294002 + OEA group), high glucose and oxygen-glucose deprivation/reperfusion and CPZ treatment group (HG + OGD/R + CPZ group), and high glucose and oxygen-glucose deprivation/reperfusion and CPZ and OEA treatment group (HG + OGD/R + CPZ + OEA group). The final concentration of OEA (Sigma-Aldrich, St. Louis, MO, United States) in the treatment group was 40 μM pretreatment for 6 h. In HG-OGD/R + CPZ + OEA group and HG-OGD/R + LY294002 + OEA group, LY294002 was pretreated with a final concentration of 20 μM for 1 h and CPZ was pretreated with a final concentration of 100 μM for 30 min, and then 40 μM OEA for 6 h, followed by OGD/R treatment.

### Cell viability assay

Cell viability was assayed by CCK-8 (Yeason Co., Ltd., Shanghai, China). After trypsin digestion of H9C2 cells at the logarithmic growth phase, 5 × 10^3^ cells/well were uniformly inoculated into 96-well plates. 10 μl of CCK-8 working solution was added into each well, followed by incubation for 2 h at 37°C. Then, the absorbance was detected at 450 nm by a fluorescence microplate reader (Thermo Fisher Scientific, Waltham, MA, United States). The cells without any treatment were used as the control group, and the relative cell viability was calculated as follows: cell relative viability = [(A − C)/(B − C)], where A is the optical density (OD) value in the experimental group, B represents the OD value in the control group, and C is the OD value in the blank group.

### Immunofluorescence assay

The H9C2 cells at the logarithmic growth phase were uniformly inoculated into 24-well plates with glass crawl sheets that were placed in advance in the plates, and 4% paraformaldehyde solution was added to fix the cells for 15 min. After being blocked with 10% goat serum, the primary antibody reaction solution (TRPV1, 1:400, abcam, ab203103) diluted with phosphate-buffered saline with Tween-20 (PBST) was added into samples, followed by incubation at 4°C overnight.

After thrice washing with PBST, the corresponding fluorescent secondary antibody (goat anti-rabbit, 488 nm, 1:300) (Yeason Co., Ltd., Shanghai, China) was added into each well and incubated for 2 h in the darkness, followed by staining with 4′,6-diamidino-2-phenylindole (DAPI) (Yeason Co., Ltd., Shanghai, China) solution (460 nm, 1:1,000). The sections were stored in a wet box and observed under a fluorescence microscope.

### Annexin V/PI apoptosis assay

The level of cell apoptosis was measured by Annexin V-PI cell apoptosis Assay kit (MeilunBio Co., Ltd., Dalian, China). According to the manufacturer’s instructions, cells were digested with trypsin (without EDTA), and collected in the corresponding centrifuge tubes at 1,000 rpm for 5 min. Subsequently, the cells were twice washed with pre-chilled PBS solution. Next, 5 μl Annexin V-FITC reagent, 10 μl PI-PE reagent, and 100 μl 1 × binding buffer were added into each tube to dilute and mix the cell suspension for 15 min. Then, the cells were filtered through a 200-mesh filter. Negative control, PI-PE single-staining positive control, and Annexin V-FITC single-staining positive control were set up to adjust the fluorescence compensation.

### Measurement of reactive oxygen species

Intracellular ROS level was detected by dichloro-dihydro-fluorescein diacetate (DCFH-DA) solution (Beyotime Institute of biotechnology, Wuhan, China). DCFH-DA was diluted with a serum-free medium according to the manufacturer’s instructions. The cells were collected and re-suspended in the DCFH-DA working solution with a density of 1 × 10^6^-2 × 10^7^ cells/ml, and incubated for 20 min at 37°C. Rosup reactive oxygen stimulant was added to the positive control cell suspension as the positive control. Then, the cells in each group were filtered through a 200-mesh filter, and a flow cytometer was used to detect the fluorescence intensity of DCF at the excitation wavelength of 488 nm.

### TUNEL assay

The terminal deoxynucleotidyl transferase dUTP nick end labeling (TUNEL) assay was used to measure the cell apoptosis. According to the manufacturer’s instructions of TUNEL assay kit (Yeason Co., Ltd., Shanghai, China), 100 μl of 1 × equilibration buffer per well was added to completely infiltrate the crawls, followed by incubation for 15 min at room temperature. Subsequently, a sufficient TdT incubation buffer was prepared using the 100 μl system required for each cell crawl. After aspirating the liquid in the wells, 100 μl TdT buffer was added and incubated for 60 min at 37°C in the darkness. Then, the cells were washed with PBS and the nuclei were stained with DAPI. The green fluorescence was detected at 520 ± 20 nm and DAPI was observed at 460 nm using a confocal fluorescence microscope (FV1000; Olympus Co., Ltd., Tokyo, Japan) with an adjusted fluorescence filter.

### Western blotting

The protein samples were added into a volume of 10 μl per well, and then, the voltage was set to 80 V for 30 min, followed by 120 V for 60 min. Subsequently, the polyvinylidene difluoride (PVDF) membranes were covered with the gel surfaces. The common molecules were wet rotated at 160 mA for 60 min, small molecules at 160 mA for 30 min, and large molecules at 80 V for 80 min, and the low temperature was maintained during the transfer process. The PVDF membranes were blocked with 5% milk powder for 2 h at room temperature. Then, the membranes were washed with TBST for 5 min and incubated with primary antibody reaction solution overnight at 4°C. The primary antibodies used in this study were mouse anti-β-Actin (1:1,000, sc-47778, Santa Cruz), mouse anti-TRPV1 (1:1,000, ab203103, Abcam)), mouse anti-PI3K-P85 (1:2000, 4G3C11, Proteintech), rabbit anti-p-Akt (1:1,000, #4060, Cell Signaling Technology), rabbit anti-cleaved-caspase-3 (1:1,000, #9664, Cell Signaling Technology), rabbit anti-Bcl-2 (1:2000, 4H8C6, ABclonal) or mouse anti-Bax (1:2000, 4G5E8, Proteintech). The membranes were thrice washed with TBST for 10 min each time, followed by incubation for 2 h with secondary antibody reaction solution. After thrice washing with TBST for 10 min each time, the membranes were placed in the IS4000R fully automated image workstation and fully infiltrated with ECL chemiluminescent solution (Yeason Co., Ltd., Shanghai, China), and the luminescent images were obtained by the automatic exposure program.

### Statistical analysis

The data were statistically analyzed using the GraphPad Prism 8.0 software (GraphPad Software Inc., San Diego, CA, United States), and were expressed as mean ± standard error of the mean (SEM). The independent-samples *t*-test was used to compare data between the two groups, and one-way analysis of variance (ANOVA) was employed to compare data among multiple groups. *p* < 0.05 was considered statistically different.

## Results

### Pretreatment with oleoylethanolamide decreased the levels of CK-MB, LDH, and MDA, while increased the level of SOD in diabetic rats

**FIGURE 1:**
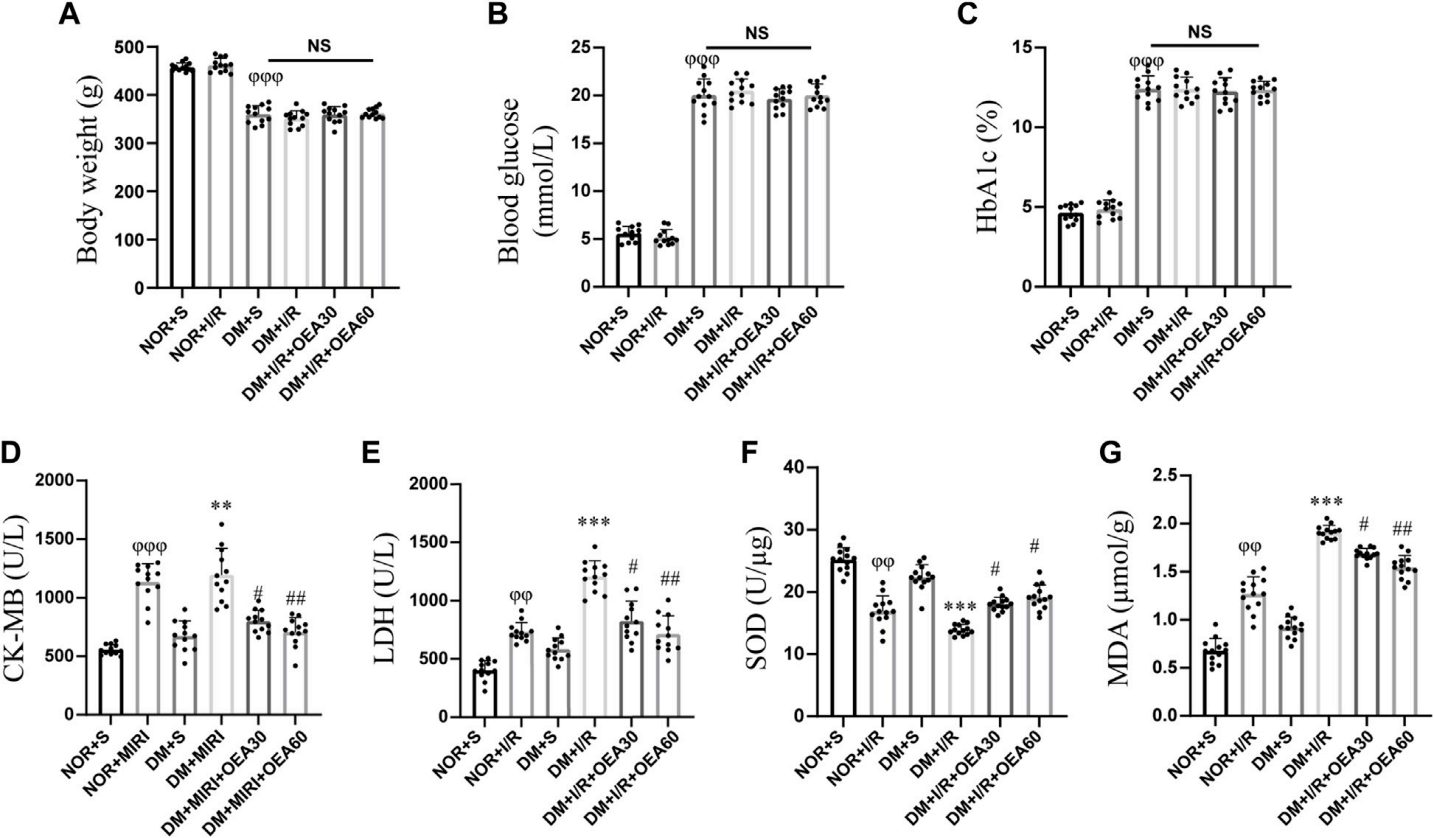
OEA decreased the levels of CK-MB, LDH, and MDA, while increased the level of SOD. **(A–C)** A myocardial ischemia-reperfusion model for diabetic rats was established, and the changes in body weight, blood glucose level, and HbA1c level in each group (*n* = 12) were measured; **(D–G)** Biochemical detection of changes in the levels of CK-MB and LDH in serum, and levels of SOD and MDA in myocardium of rats after OEA administration (*n* = 12). Data were expressed as mean ± SEM, ^φφ^ *P* <0.01, ^φφφ^ *P* <0.001 vs. NOR + S group; **p < 0.01, ***p < 0.001 vs. DM + S group; ^#^ *p* < 0.05, ^##^ *p* < 0.01 vs. DM + I/R group.

After intraperitoneal injection of STZ to establish the diabetic rat model, diabetic rats in the DM + I/R + OEA30 group and DM + I/R + OEA60 group were treated with OEA continuously for 3 days before I/R operation. As shown in Figures 1A–C, there were no significant changes in body weight, blood glucose, and HbA1c level after OEA treatment, indicating that short-term OEA treatment had no therapeutic effect on diabetic rats. Next, the effects of OEA on the levels of CK-MB, LDH, SOD, and MDA were analyzed. The levels of CK-MB and LDH were elevated during MIRI. The biochemical test results showed that the levels of CK-MB and LDH were markedly reduced in the DM + I/R + OEA30 group and DM + I/R + OEA60 group compared with those in the DM + I/R group (Figures 1D,E). Furthermore, SOD plays a protective role in oxidative stress of myocardial tissue, and MDA reflects the level of oxidative stress. Pretreatment with OEA could increase the level of SOD (U/μg) and decrease the level of MDA in the myocardial tissue of diabetic rats (Figures 1F,G).

### Oleoylethanolamide attenuated myocardial tissue injury in diabetic myocardial I/R rats

**FIGURE 2:**
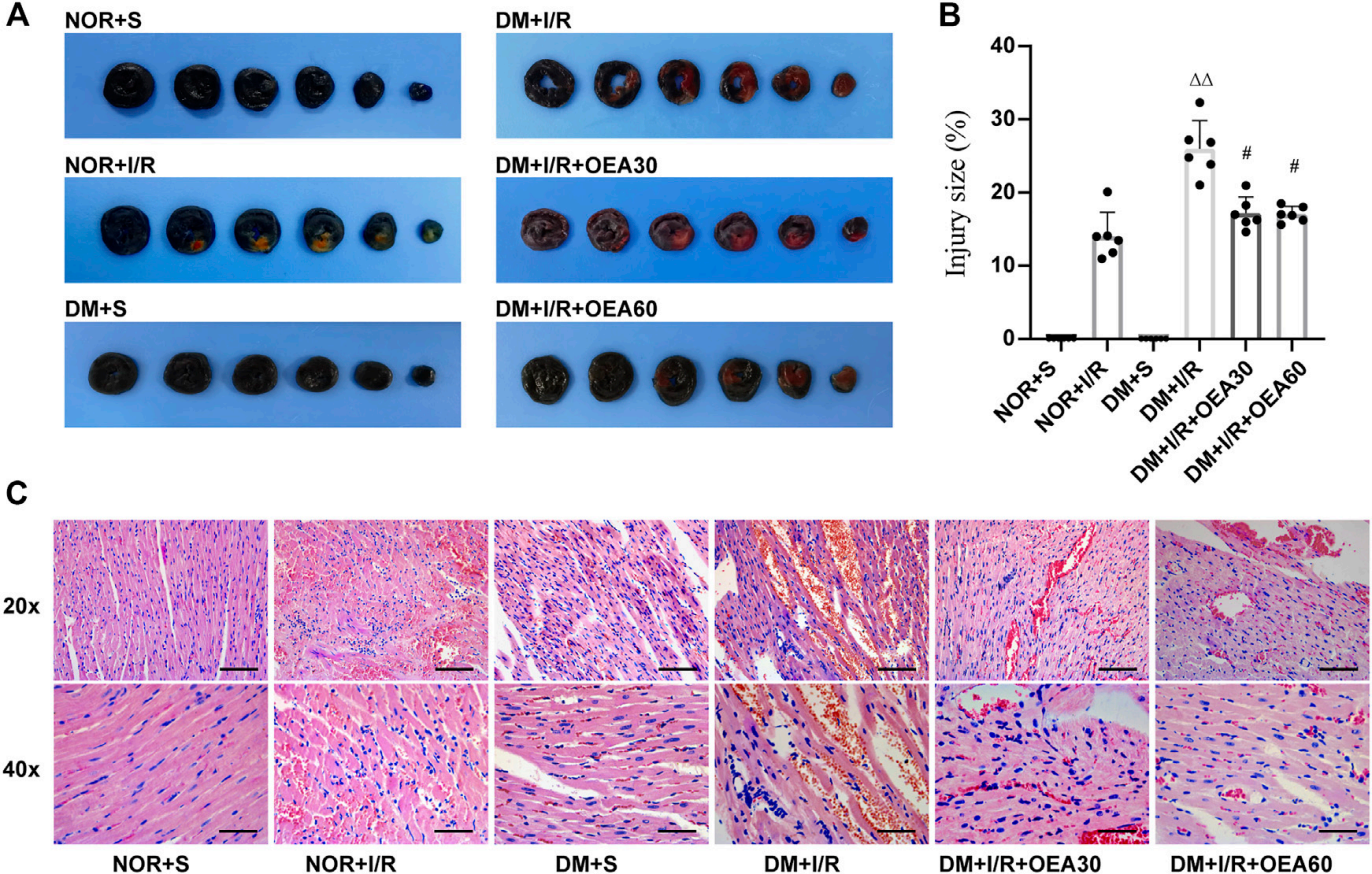
OEA attenuated myocardial tissue injury in diabetic myocardial ischemia-reperfusion rats. **(A,B)** Evans blue-TTC staining of myocardium, in which blue represents the region of healthy myocardial tissue, and red represents the region of ischemia-reperfusion injury (*n* = 6); **(C)** HE staining of myocardial tissue, including hematoxylin (blue) staining of nucleus, and eosin (red) staining of cytoplasm, 20x scale = 100 μm, 40x scale = 50 μm. Data were expressed as mean ± SEM, ^ΔΔ^ *P* < 0.01 vs. NOR + I/R group; ^#^ *p* < 0.05 vs. DM + I/R group.

The infarcted area of myocardial tissue was assessed using Evans blue and TTC staining (Figures 2A,B). Compared with the control group, the DM + I/R group showed a significantly enlarged myocardial infarction size, while pretreatment with OEA reduced the size of myocardial infarction. The pathological changes evidenced by H&E staining (Figure 2C) in the DM + I/R group indicated severe myocardial fiber rupture, disordered tissue structure, and atrophy and aggregation of nuclei. In the DM + I/R + OEA30 and DM + I/R + OEA60 groups, the degree of tissue fibrosis and cell injury were significantly improved. These results suggested that OEA had a protective effect on myocardial tissue injury in diabetic myocardial I/R rats.

### Protective effects of oleoylethanolamide on H9C2 cells treated with high glucose and OGD/R

**FIGURE 3:**
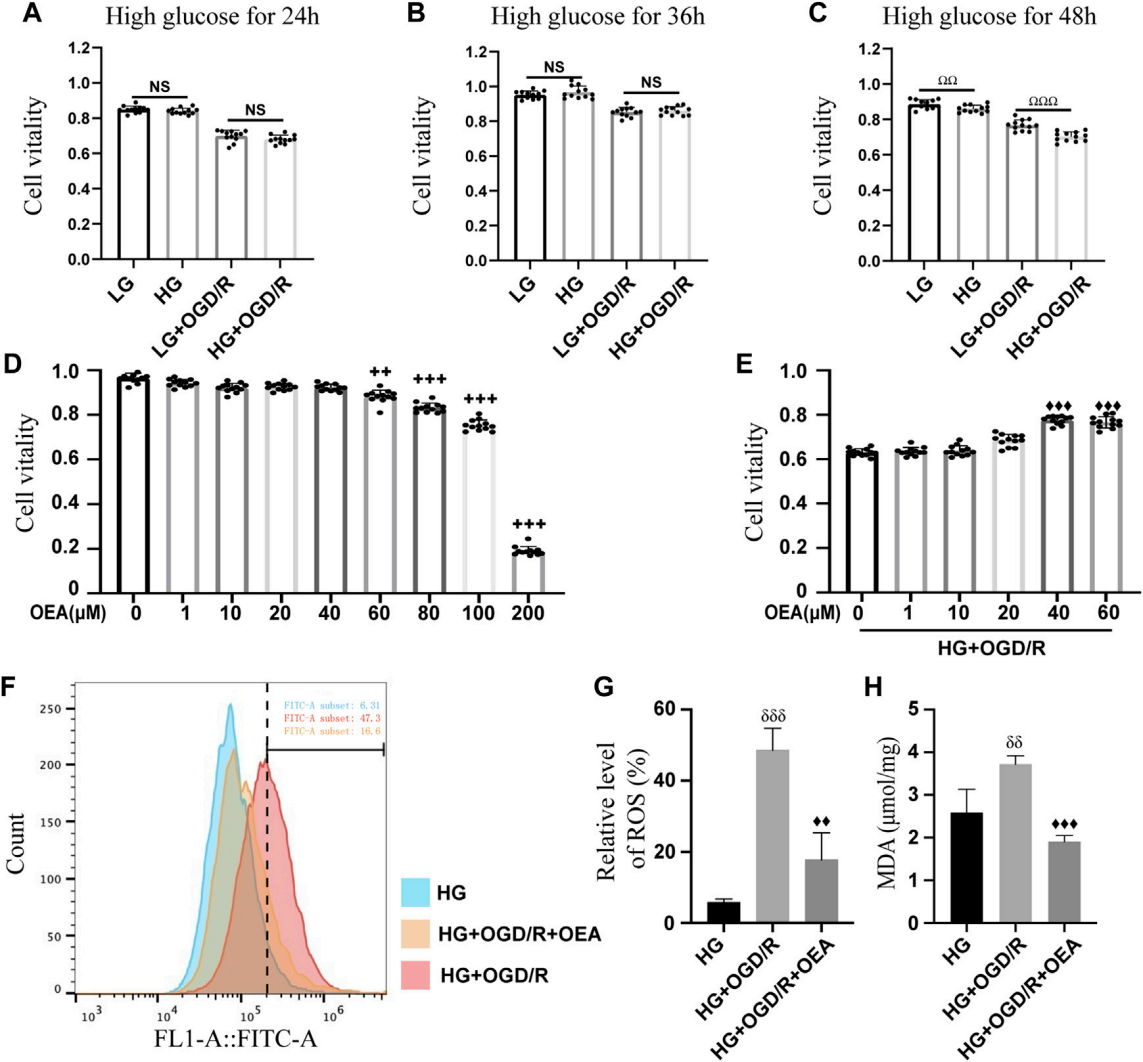
*In vitro* protective effects of OEA on the model. **(A–C)** CCK-8 assay results showed the viability of H9C2 cells treated with OGD/R at different time points after high glucose induction (*n* = 12). **(D)** The CCK-8 assay data revealed the viability of H9C2 cells after 24 h of treatment with different concentrations of OEA (*n* = 6). **(E)** The viability of H9C2 cells was analyzed by CCK-8 assay after treatment with different concentrations of OEA *in vitro* with high glucose and OGD/R (*n* = 12). **(F,G)** The ROS level in each group after OGD/R and OEA (40 μM) treatment was detected by flow cytometry (*n* = 3). **(H)** MDA level in each group after high glucose and OGD/R and OEA (40 μM) treatment (*n* = 3). Data were expressed as mean ± SEM, ^ΩΩ^ *P* < 0.01, ^ΩΩΩ^ *P* < 0.001, the control group was shown in the horizontal line label; ^++^ *p* < 0.01, ^+++^ *p* < 0.001 vs. OEA 0 μM group; ^♦♦^ *p* < 0.01, ^♦♦♦^ *p* < 0.001 vs. HG + OGD/R group; ^δδ^ *P* < 0.01, ^δδδ^ *P*< 0.001 vs. HG group.

H9C2 cells were pretreated with high glucose at different time points (24, 36, and 48 h) before OGD/R. The results of CCK-8 assay (Figures 3A–C) showed that high glucose induction for 48 h could significantly reduce the viability of H9C2 cells, thus, high glucose treatment for 48 h was used as the experimental condition in the present study. Subsequently, OEA toxicity test and drug concentration screening test were performed on H9C2 cells. The CCK-8 data indicated that OEA concentration above 80 μM could affect the viability of H9C2 cells (Figure 3D). After 48 h of high glucose induction, OGD for 6 h, and reperfusion for 4 h, 40 μM and 60 μM OEA had positive protective effects on H9C2 cells, and there was no significant difference in cell viability between the two dose-dependent groups (Figure 3E). Therefore, 40 μM concentration was selected as the OEA treatment condition *in vitro*. According to the measurement of ROS (Figures 3F,G), compared with HG group, the ROS levels of H9C2 cells were significantly increased in HG + OGD/R treatment. However, ROS level was dramatically decreased after OEA treatment. As shown in Figure 3H, OEA could effectively reduce the MDA level of H9C2 cells after high glucose and OGD/R treatment. The above-mentioned results demonstrated that OEA could alleviate the damage to H9C2 cells induced by high glucose and OGD/R treatment.

### Oleoylethanolamide increased the expression level of TRPV1 in diabetic rats with MIRI

**FIGURE 4:**
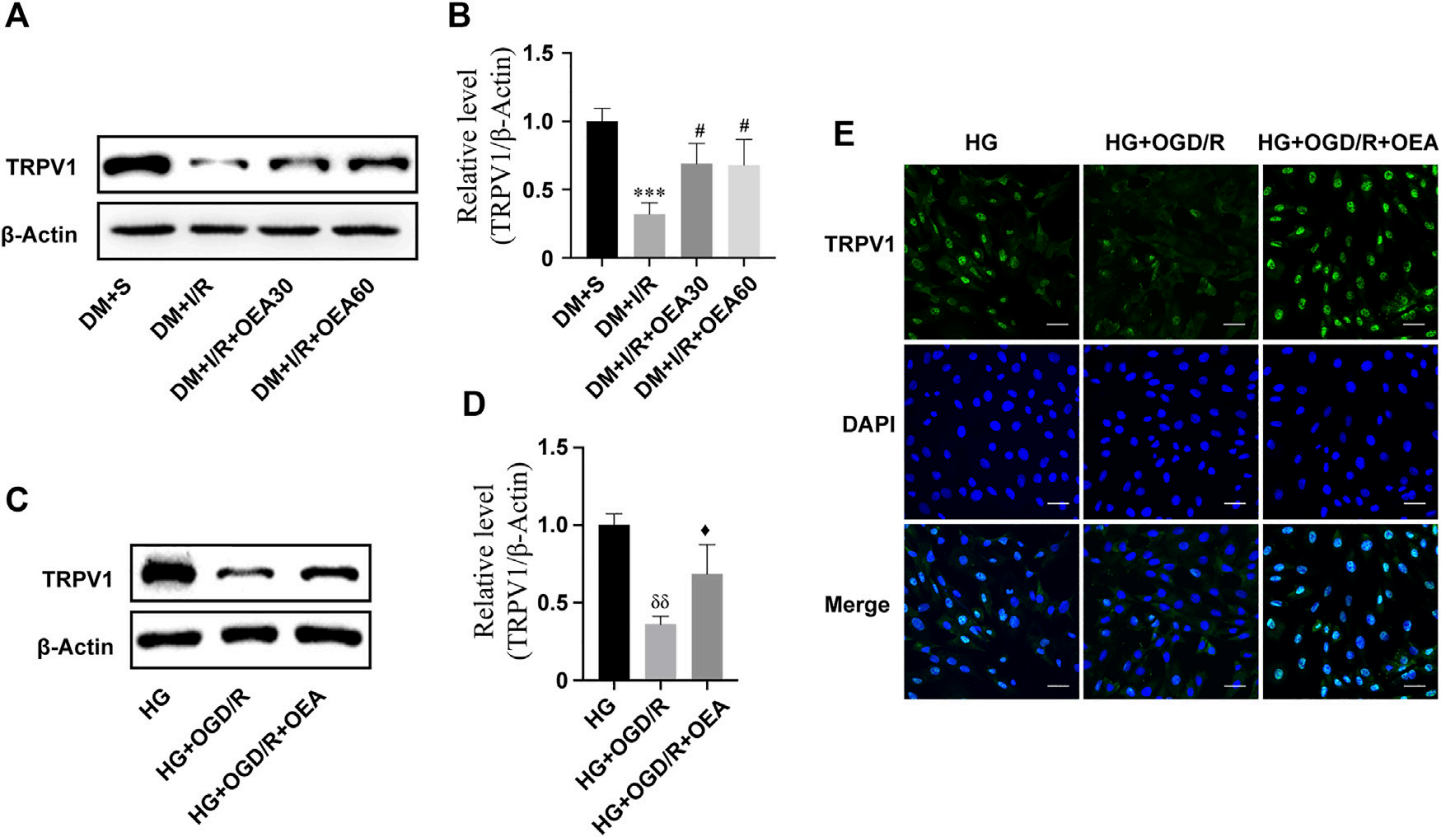
The expression level of TRPV1 was upregulated after OEA treatment. **(A,B)** Semi-quantitative analysis revealed that OEA pretreatment reduced the expression level of TRPV1 protein in diabetic myocardial ischemia-reperfusion injury rats. **(C,D)** Semi-quantitative analysis indicated that OEA pretreatment decreased the expression level of TRPV1 protein in H9C2 cells. **(E)** IF results showed immunofluorescence staining of cells in each group with DAPI fluorescent-labeled dye (nucleus, blue) and TRPV1 (green), scale = 50 μm. Data were expressed as mean ± SEM, ***p < 0.001 vs. DM + S group; ^#^ *p* < 0.05 vs. DM + I/R group; ^δδ^ *P* <0.01 vs. HG group; ^♦^ *p* < 0.05 vs. HG + OGD/R group.

The results of WB showed that the expression level of TRPV1 in rat myocardium significantly decreased after MIRI (Figures 4A,B). Pretreatment with OEA (30 and 60 mg/kg) could increase the expression level of TRPV1 in diabetic rats with MIRI (Figures 4A,B). In addition, the expression of TRPV1 was greatly down-regulated in H9C2 cells treated with high glucose or OGD/R (Supplementary Figures S1A,B). OEA also upregulated the expression level of TRPV1 in H9C2 cells induced by high glucose and OGD/R (Figures 4C,D). Similar results were obtained by the TRPV1 immunofluorescence staining of H9C2 cells (Figure 4E).

### Oleoylethanolamide activated the PI3K/Akt signaling pathway and attenuated I/R-induced myocardial apoptosis in diabetic rats

**FIGURE 5:**
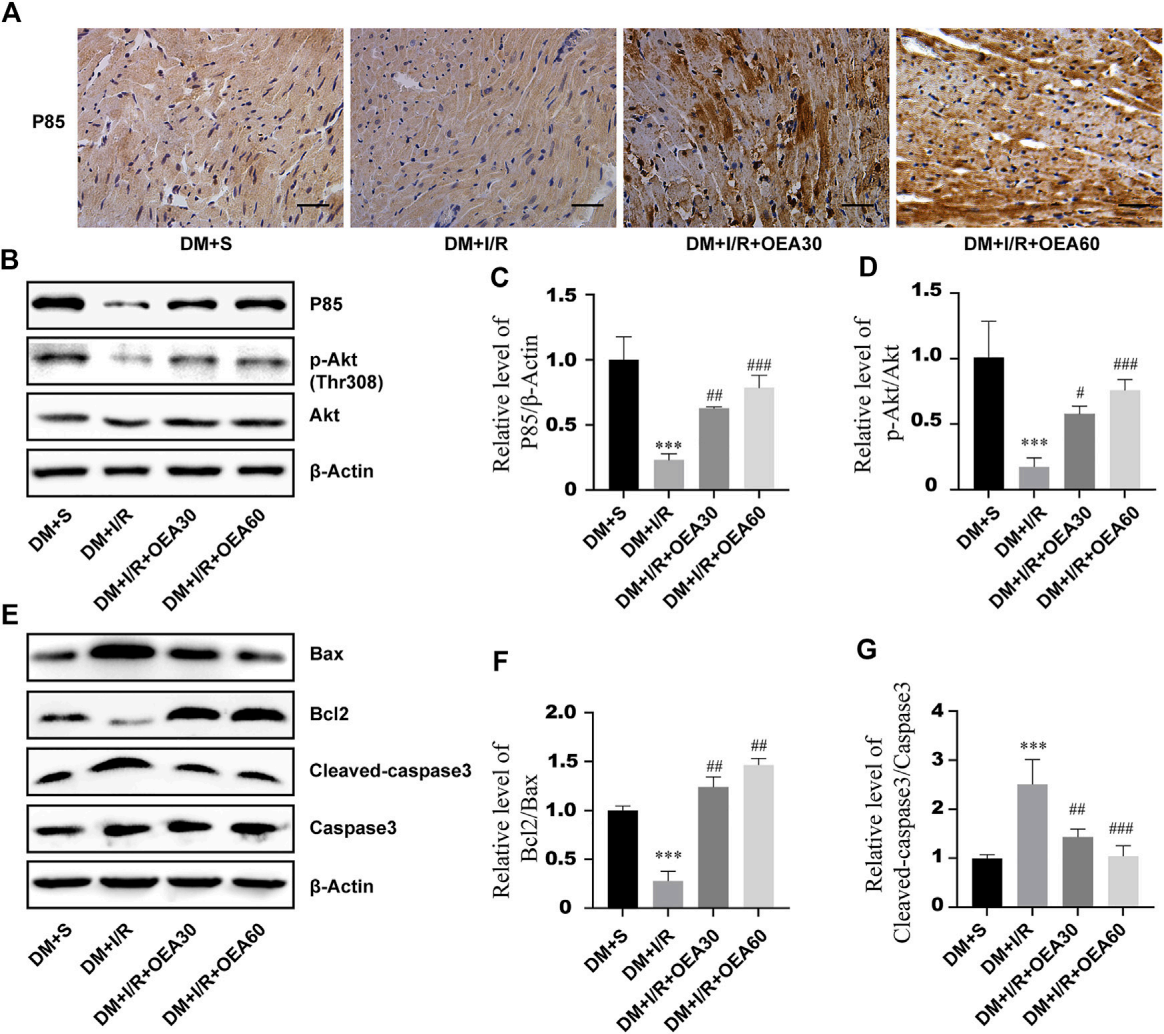
OEA activated the PI3K/Akt signaling pathway and downregulated the expression levels of apoptosis-related proteins. **(A)** The expression level of P85 in rat myocardium was detected by immunohistochemistry, scale = 50 μm. **(B–G)** WB was used to detect the expression levels of P85, p-Akt, Bax, Bcl-2 and cleaved caspase-3 proteins in myocardial tissues of rats in each group. Data were expressed as mean ± SEM, ***p < 0.001 vs. DM + S group; ^#^ *p* < 0.05, ^##^ *p* < 0.01, ^###^ *p* < 0.001 vs. DM + I/R group.

In order to clarify the effects of OEA on the PI3K/Akt signaling pathway and apoptosis-related proteins, IHC and WB were used to detect the expression levels of apoptosis-related proteins in myocardial tissue of diabetic rats. Compared with the DM + I/R group, the catalytic subunit P85 of PI3K was significantly overexpressed in the myocardial tissue in the DM + I/R + OEA pretreatment group (Figure 5A), and Figures 5B,C confirmed this finding. As an important marker of activation of the PI3K/Akt pro-survival signaling pathway, the expression level of p-Akt conspicuously increased after OEA pretreatment (Figures 5B,D). By detecting the expression levels of downstream apoptosis-related proteins, the results showed that the expression levels of cleaved caspase-3 were significantly decreased, while the level of Bcl-2/Bax was dramatically increased after OEA treatment (Figures 5B–G). These results demonstrated that OEA could activate the PI3K/Akt signaling pathway and downregulate the expression levels of apoptosis-related proteins in myocardial tissue of I/R-induced diabetic rats.

### Pretreatment with oleoylethanolamide attenuated OGD/R-induced apoptosis of H9C2 cells under high glucose conditions

**FIGURE 6:**
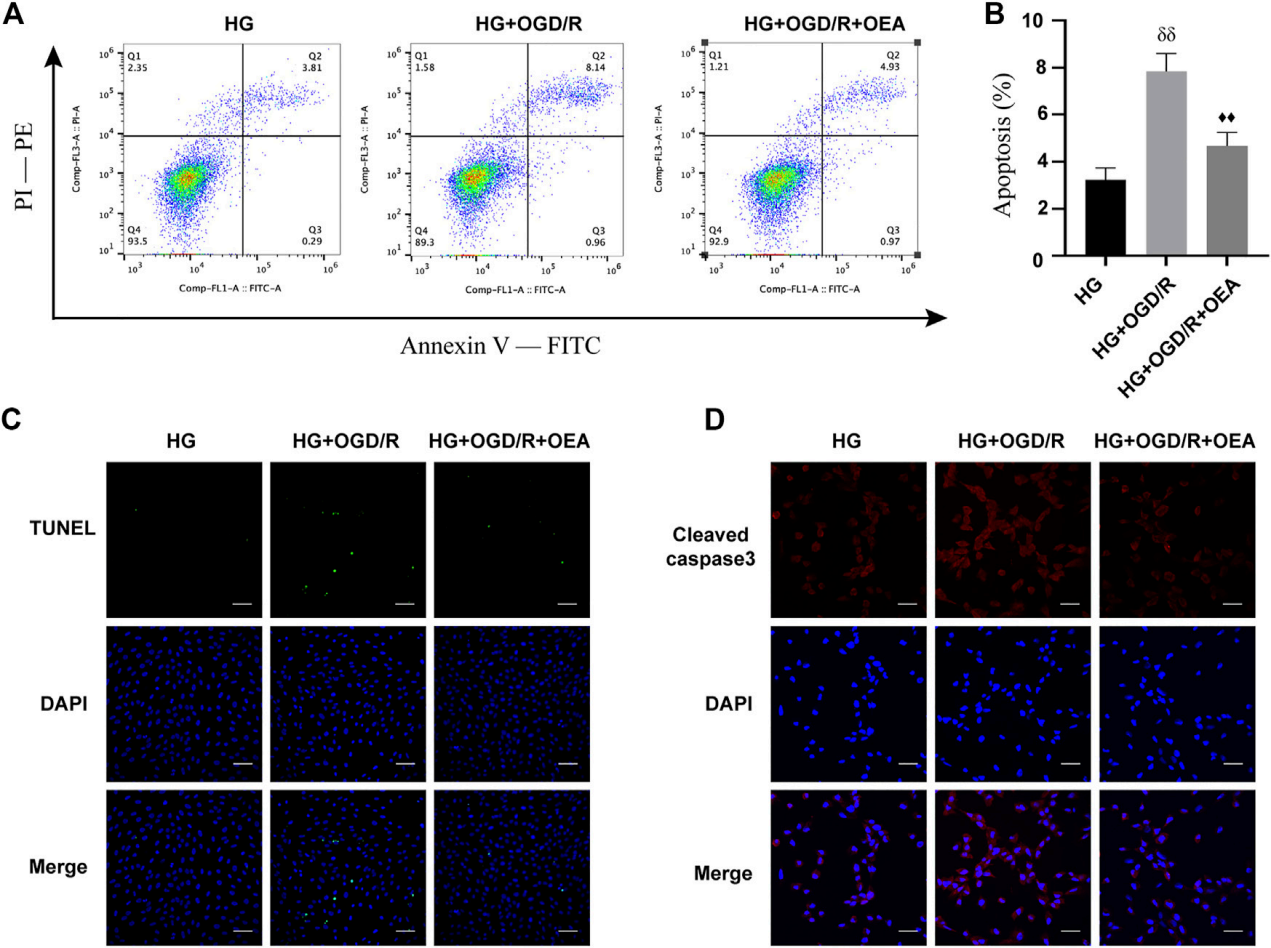
OEA inhibited the apoptosis of H9C2 cells after OGD/R induced by high glucose. **(A)** Flow cytometry was performed on each group of cells using FITC/PE channel. Annexin V fluorescence intensity was represented on the horizontal axis. PI fluorescence intensity was represented on the vertical axis, and Q2 region represents the number of apoptotic cells. **(B)** Statistical analysis of apoptotic cells was carried out by flow cytometry (*n* = 3). **(C)** DAPI fluorescent dye (nucleus, blue) and TUNEL (green) were used for immunofluorescence staining of cells in each group, scale = 100 μm. **(D)** DAPI fluorescent dye (nucleus, blue) and cleaved caspase-3 (red) were used for immunofluorescence staining of cells in each group, scale = 50 μm. Data were expressed as mean ± SEM, ^δδ^ *P* <0.01 vs. HG group, and ^♦♦^ *p* < 0.01 vs. HG + OGD/R group.

The effects of OEA on OGD/R injury of H9C2 cells under high glucose conditions were further determined by detecting apoptosis of H9C2 cells. According to the results of Annexin V-FITC/PI-PE assay, apoptosis level was significantly elevated in the HG + OGD/R group compared with that in the HG control group, while apoptosis level in the HG + OGD/R + OEA pretreatment group was significantly reduced (Figures 6A,B). Similarly, according to the results of the TUNEL assay (Figure 6C), compared with the HG + OGD/R group, the apoptosis level in the HG + OGD/R + OEA group significantly decreased. Subsequently, immunofluorescence staining was performed on each group of cells, and it was revealed that OEA pretreatment could markedly reduce the expression level of cleaved caspase-3 (Figure 6D). These results suggested that OEA could inhibit the apoptosis of H9C2 cells induced by OGD/R after high glucose treatment.

### Oleoylethanolamide inhibited apoptosis of H9C2 cells induced by high glucose and I/R injury through activating the PI3K/Akt signaling pathway

**FIGURE 7:**
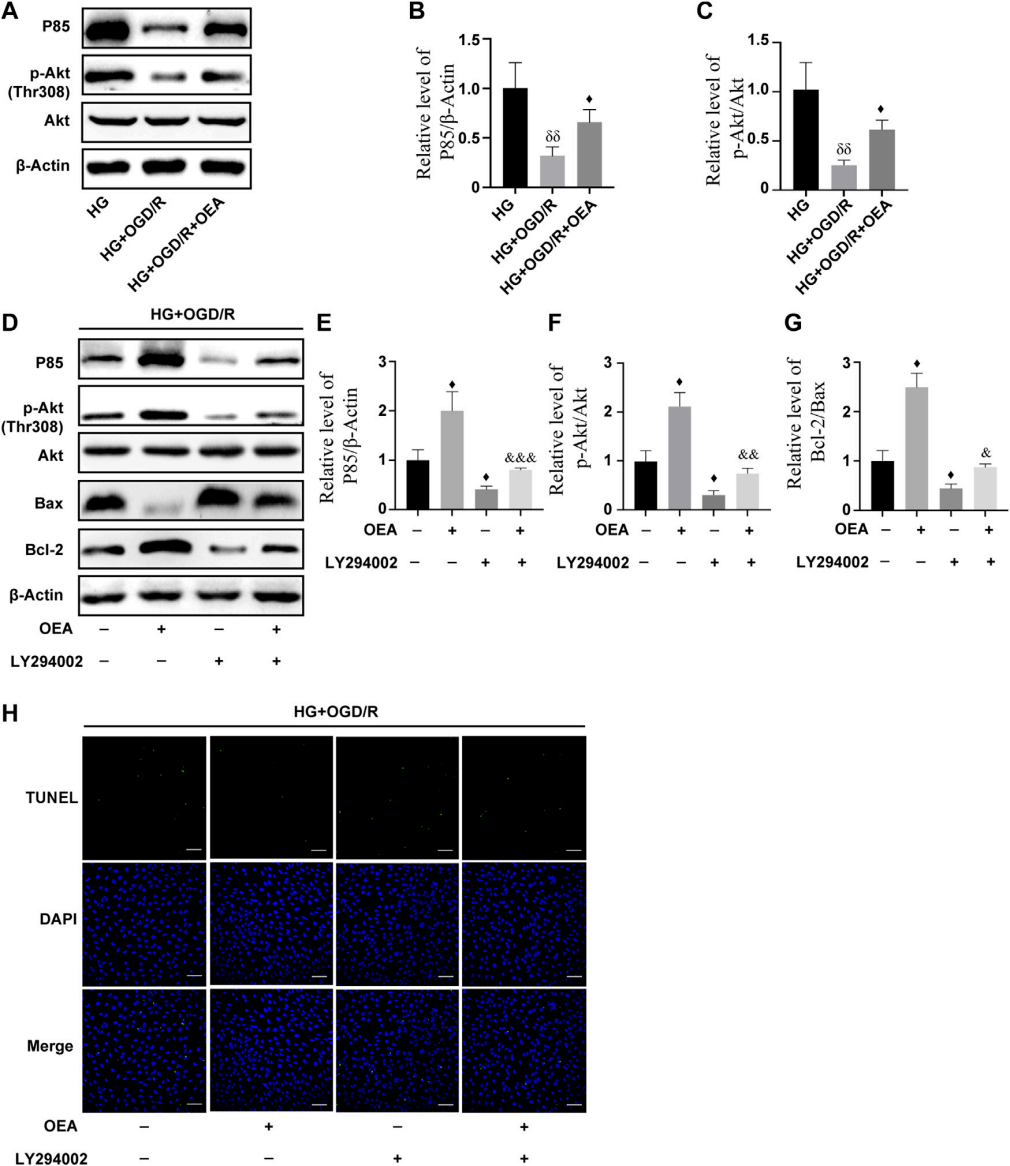
OEA inhibited high glucose and OGD/R-induced apoptosis of cells by regulating the PI3K/Akt signaling pathway. **(A–C)** The expression levels of P85 and P-Akt in cardiomyocytes induced by high glucose and OGD/R and pretreated with OEA. **(D–G)** The expression levels of P85, P-Akt, and Bax, Bcl-2 in cells induced by high glucose and OGD/R and pretreated with OEA or LY294002. **(H)** DAPI fluorescent dye (nucleus, blue), TUNEL (green) apoptotic markers were used for immunofluorescence staining of cells in each group, scale = 100 μm. Data were expressed as mean ± SEM, ^δδ^ *P* < 0.01 vs. HG group; ^♦^ *p* < 0.05 vs. HG + OGD/R group; and ^&^ *p* < 0.05, ^&&^ *p* < 0.01, ^&&&^ *p* < 0.001 vs. HG + OGD/R + LY294002 group.

According to the results of WB, compared to the control group, the expression levels of P85 and p-Akt in H9C2 cells were markedly downregulated after high glucose and OGD/R treatment (Supplementary Figures S3A–D), while the expression levels were significantly upregulated in the OEA pretreatment group (Figures 7A–C). To further explore whether PI3K/Akt is the main protective pathway of OEA, PI3K inhibitor, LY294002, was used to pretreat cells before OGD/R to inhibit P85 expression level. The results showed that OEA could upregulate the expression levels of P85 and p-Akt proteins inhibited by LY294002 (Figures 7D–F). In addition, LY294002 pretreatment notably improved the expression of pro-apoptotic protein (Bax) and reduced the expression level of anti-apoptotic protein (Bcl-2), while co-treatment of OEA with LY294002 diminished the expression of Bax and elevated the expression level of Bcl-2 compared with that in the LY294002 pretreatment group (Figures 7D–G). As shown in Figure 7H, the apoptosis level of cells was increased after LY294002 treatment, and compared with the LY294002 group, the apoptosis level in the LY294002 + OEA group markedly decreased. The above-mentioned results demonstrated that OEA could play a protective role against apoptosis under high glucose and OGD/R conditions by activating the PI3K/Akt signaling pathway.

### Oleoylethanolamide regulated the PI3K/Akt signaling pathway through activation of TRPV1

**FIGURE 8:**
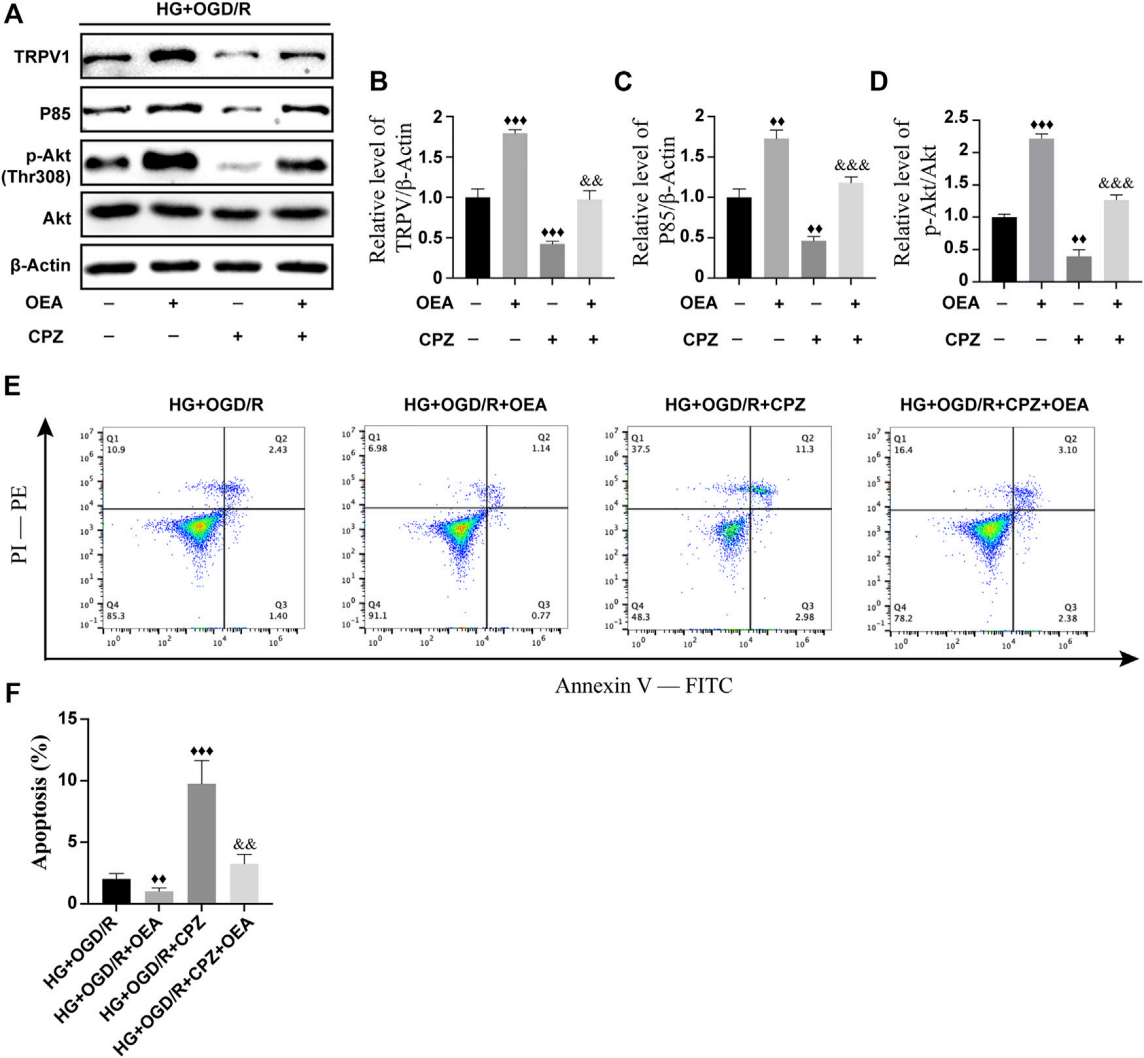
OEA regulated the PI3K/Akt signaling pathway through activation of TRPV1. **(A–D)** WB results showed the expression levels of TRPV1, P85, and p-AKT in H9C2 cells induced by high glucose and OGD/R and treated with OEA or CPZ. **(E)** Analysis of cells in each group was performed by flow cytometry. Horizontal axis represents Annexin V fluorescence intensity. Vertical axis represents PI fluorescence intensity, and Q2 region represents the number of apoptotic cells. **(F)** Statistical analysis of apoptotic cells was performed by flow cytometry (*n* = 3). Data were expressed as mean ± SEM, ^♦♦^ *p* < 0.01, ^♦♦♦^ *p* < 0.001 vs. HG + OGD/R group; and ^&&^ *p* < 0.01, ^&&&^ *p* < 0.001 vs. HG + OGD/R + CPZ group.

To further investigate whether OEA can act as an agonist of TRPV1 and then regulate the PI3K/Akt signaling pathway, H9C2 cells were pretreated with CPZ, a receptor antagonist of TRPV1, prior to OEA pretreatment. As shown in Figures 8A–D, the expression level of TRPV1 was significantly inhibited after CPZ treatment, and the expression levels of P85 and p-Akt were downregulated. However, the inhibitory effects of CPZ on the expression levels of TRPV1, P85, and p-Akt were attenuated in the OEA and CPZ co-treatment group. Meanwhile, as illustrated in Figures 8E,F, the apoptosis level was markedly elevated after CPZ treatment compared with that in the OEA-treated group, whereas the combined treatment of OEA and CPZ could downregulate the increase of apoptosis caused by CPZ. These findings demonstrated that OEA could regulate the PI3K/Akt signaling pathway by activating TRPV1, thereby inhibiting apoptosis of cardiomyocytes.

## Discussion

The mechanism of MIRI is extremely complex. To date, some drugs have been shown to be effective in alleviating MIRI, while preventive and therapeutic methods have accompanied by poor efficacy for patients with myocardial ischemia complicated with diabetes. OEA has been proved to be an endogenous monounsaturated lipid molecule with multiple biological functions (R Pandey et al., 2009; Gonzalez-Aparicio et al., 2014; Hill et al., 2009; Lueneberg et al., 2011; Mazzola et al., 2009). The receptors for OEA include PPARα, TRPV1 and GPR119. GPR119 is mainly expressed in islet β cells and PP cells in pancreatic tissue. OEA can activate GPR119, which is mainly related to lipid metabolism and insulin secretion, etc. (Higuchi et al., 2020; Zhao et al., 2021). OEA regulates inflammatory response and neuroprotection through activating PPARα, while TRPV1 is related to cell proliferation and survival. The study found that OEA significantly attenuated dox-induced oxidative stress in the heart and apoptosis *in vitro* and *in vivo*. These therapeutic effects could be blocked by capsaicin, a TRPV1 antagonist, but not by GW6471, an antagonist of PPARα (Qin et al., 2022). Ge et al. (2016) reported that dox-induced cardiac deficits and apoptosis could be reversed by SA13353, a TRPV1 agonist. Meanwhile, Jiang et al. (2018) found that when TRPV1^−/−^, apoptosis levels were significantly increased and infarct size was increased in the myocardium of mice with MIRI (Buch et al., 2018; Jiang et al., 2018; Zhai et al., 2020). A previous study performed by our research team indicated that OEA could play a hypoglycemic role by regulating hepatic glycogen synthesis and gluconeogenesis, thereby ameliorating diabetes (Ren et al., 2020). In addition, OEA has a protective effect on acute cerebral ischemia (Zhou et al., 2012; Zhou et al., 2017). However, the application of OEA for I/R injury in diabetic conditions and its underlying mechanisms have not been fully clarified. In the present study, we attempted to indicate whether OEA could improve MIRI in diabetic rats. The results showed that OEA pretreatment could attenuate the myocardial injury in diabetic rats induced by I/R, and also reduce the myocardial cell apoptosis induced by high glucose and OGD/R. Additionally, it was revealed that OEA pretreatment could activate TRPV1 *in vitro* and *in vivo*, which in turn activated the PI3K/Akt signaling pathway. CPZ or blocking the PI3K/Akt signaling pathway could significantly attenuate the protective effect of OEA against high glucose and OGD/R-induced apoptosis in cardiomyocytes. These studies suggested that OEA could ameliorate MIRI in diabetic conditions by activating TRPV1 to regulate the PI3K/Akt signaling pathway.

In the present study, a diabetic rat model of MIRI was established using high-fat feeding and STZ injection-induced diabetes combined with ligation-reflow left anterior descending coronary artery surgery. The data showed that the diabetic model induced by STZ successfully exhibited weight loss, hyperglycemia, and elevated HbA1c level. In this research, compared with non-diabetic rats, MIRI was exacerbated and myocardial infarcted area was enlarged in diabetic rats. These findings further confirmed that diabetes could aggravate MIRI. The results of this experiment indicated that OEA pretreatment decreased the levels of CK-MB, LDH, and MDA, while increased SOD level in diabetic rats with MIRI. Meanwhile, OEA markedly reduced the size of myocardial infarction in diabetic rats and improved the structural damage and morphological changes of cardiomyocytes. The model of H9C2 cells induced by high glucose combined with OGD/R was established *in vitro*. The analysis revealed that OEA could effectively inhibit the ROS caused by OGD/R in cardiomyocytes under high glucose conditions, and upregulate MDA level. The above-mentioned results suggested that OEA can play a protective role in diabetics complicated with MIRI and alleviate oxidative stress in myocardial cells.

It has been shown that apoptosis is involved in the pathophysiological process of MIRI under diabetic conditions (Cai et al., 2002). Additionally, a previous study demonstrated that capsaicin could improve mitochondrial dysfunction and alleviate apoptosis of cardiac myocytes through activation of TRPV1 (Xiong et al., 2016). In the present study, we found that compared with non-diabetic rats, the expression level of TRPV1 in myocardial tissue of diabetic rats with MIRI was significantly reduced, while OEA could upregulate this expression level. The same effect was observed in high glucose and OGD/R-induced H9C2 cells. Meanwhile, OEA treatment could inhibit apoptosis of H9C2 cells induced by OGD/R after high glucose treatment.

The PI3K/Akt signaling pathway is one of the important signal transduction pathways, regulating numerous cellular activities *in vivo*. The main functions of this pathway include promoting cell proliferation, inhibiting apoptosis, and modulating tissue inflammation. It was found that activation of the PI3K/Akt signaling pathway is essential to inhibit myocardial cell apoptosis during MIRI (Guan et al., 2020; Han et al., 2020; Xin et al., 2020; Tong et al., 2021). The pro-apoptotic protein Bax, as well as the anti-apoptotic protein Bcl-2, are involved in endogenous apoptosis (Cheng et al., 2018), and both apoptotic mechanisms lead to the activation of caspase-3, ultimately mediating apoptosis through nuclear activity. We, in the present study, attempted to indicate whether OEA can activate the PI3K/Akt signaling pathway to suppress apoptosis of cardiomyocytes. The results showed that OEA could upregulate the expression levels of P85 (the catalytic subunit of PI3K) and p-Akt in myocardial tissues. Similarly, this effect was confirmed in high glucose and OGD/R model of H9C2 cells. In addition, OEA notably decreased the expression levels of Bax and cleaved caspase-3 in myocardial tissue of I/R-induced diabetic rats. Consistent with the above-mentioned results, OEA inhibited OGD/R-induced activation of caspase-3 in H9C2 cells. Further research demonstrated that OEA had an antagonistic effect on the downregulation of the expression levels of P85, p-Akt, and Bcl-2 caused by the PI3K inhibitor (LY294002). Therefore, we verified that OEA could affect cardiomyocyte apoptosis by regulating the PI3K/Akt signaling pathway in diabetic rats with MIRI.

It was indicated that in the prevention and treatment of diabetic MIRI, short-term insulin treatment also failed to restore the mechanism of cardiac protection (Ebel et al., 2003; Hassouna et al., 2006). Importantly, a previous study performed by our research team showed that continuous administration of OEA for more than 2 months was efficacious in reducing blood glucose level in diabetic rats (Ren et al., 2020), whereas in the present study, short-term pre-administration of OEA into diabetic rats at 3 days before MIRI did not dramatically downregulate the levels of blood glucose and HbA1c. This suggests that short-term OEA pretreatment may not improve diabetes mellitus by regulating blood glucose level, thereby playing a cardioprotective role in MIRI. Another research showed that compared with non-diabetic rats, the decrease of TRPV1 expression level in myocardium of diabetic rats after myocardial ischemia could lead to the loss of cardioprotection function and impair myocardial function after ischemia treatment (Moll-Kaufmann et al., 1998). According to the results of the present study, when CPZ, the antagonist of TRPV1, was used *in vitro*, the expression levels of P85 and p-Akt were suppressed, while co-treatment with OEA and CPZ partially restored the expression levels of P85 and p-Akt. Similarly, administration of CPZ significantly reduced the protective effect of OEA on apoptosis, and OEA, as an agonist of TRPV1, noticeably weakened the inhibitory effect of CPZ. Therefore, it can be further clarified that OEA alleviates diabetic I/R-induced myocardial injury by activating the PI3K/Akt signaling pathway, and this function may be achieved through TRPV1.

## Conclusion

In summary, the results of the present study confirmed the cardioprotective effects of OEA, as a TRPV1 agonist, on diabetic rats with MIRI, and suggested that OEA could inhibit apoptosis through activation of the TRPV1 to regulate the PI3K/Akt signaling pathway, thereby protecting diabetic rats against I/R-induced myocardial injury. The findings may assist clinicians in developing potential therapeutic targets for alleviating MIRI in diabetic patients.

## Data Availability

The original contributions presented in the study are included in the article/Supplementary Material, further inquiries can be directed to the corresponding authors.

## Supplementary material

SUPPLEMENTARY FIGURE S1
**(A)** Anatomical sketch of coronary artery ligation in rats. The black line represents the location of coronary artery ligation. AA, aortic arch; LCA, left coronary artery. **(B)** Schematic diagram of experimental group and administration scheme in rats. I, ischemia; R, reperfusion. **(C,D)** Schematic diagram of H9C2 cell administration scheme. OGD, oxygen-glucose deprivation; R, reperfusion.

SUPPLEMENTARY FIGURE S2 Establishment of a rat model of diabetic myocardial ischemia-reperfusion injury. Rats were fed with high-fat diet for 8 weeks and received STZ for 72 h. **(A)** Weight of rats in DM group and NOR group (*n* = 12). **(B)** Comparison of fasting blood glucose level between DM group and NOR group (*n* = 12). **(C)** Glycosylated hemoglobin of rats in DM group compared with NOR group (*n* = 12). **(D)** ST segment changes of electrocardiogram before ischemia, after ischemia, and after reperfusion in I/R group. Data were expressed as mean ± SEM, ****P* <0.001 vs. NOR group.

SUPPLEMENTARY FIGURE S3 The WB results of TRPV1, P85 and p-Akt protein levels in H9C2 cells induced by high glucose and OGD/R and pretreated with OEA. **(A-B)** Compared with the control group, the expression levels of TRPV1, P85, and p-Akt in HG group and HG + OGD group was significantly decreased. Data were expressed as mean ± SEM, ^▲^ *P* < 0.01, ^▲▲^ *P* < 0.001 vs. control group.
