# Supplementary figures and images for: OEA alleviates apoptosis in diabetic rats with myocardial ischemia/reperfusion injury by regulating the PI3K/Akt signaling pathway through activation of TRPV1
Source: Front Pharmacol. 2022 Nov 14;13:964475. doi: 10.3389/fphar.2022.964475 (PMC9701823; doi:10.3389/fphar.2022.964475)

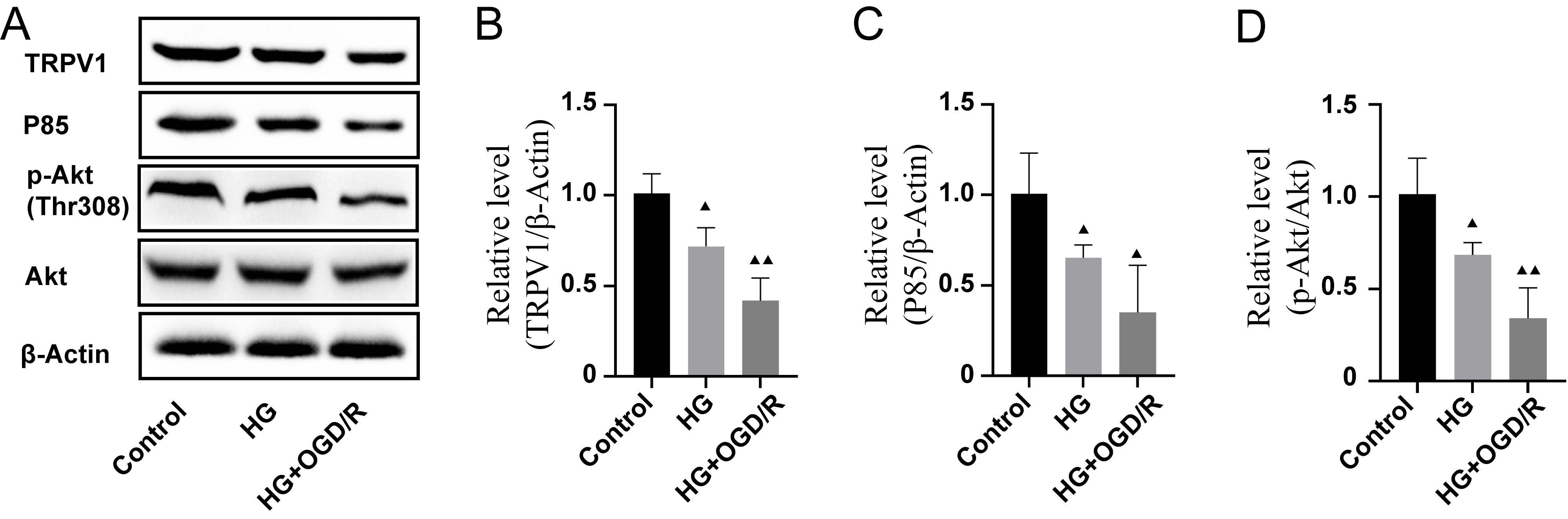

Supplement: Supplementary file 1 [file Image3.TIF]

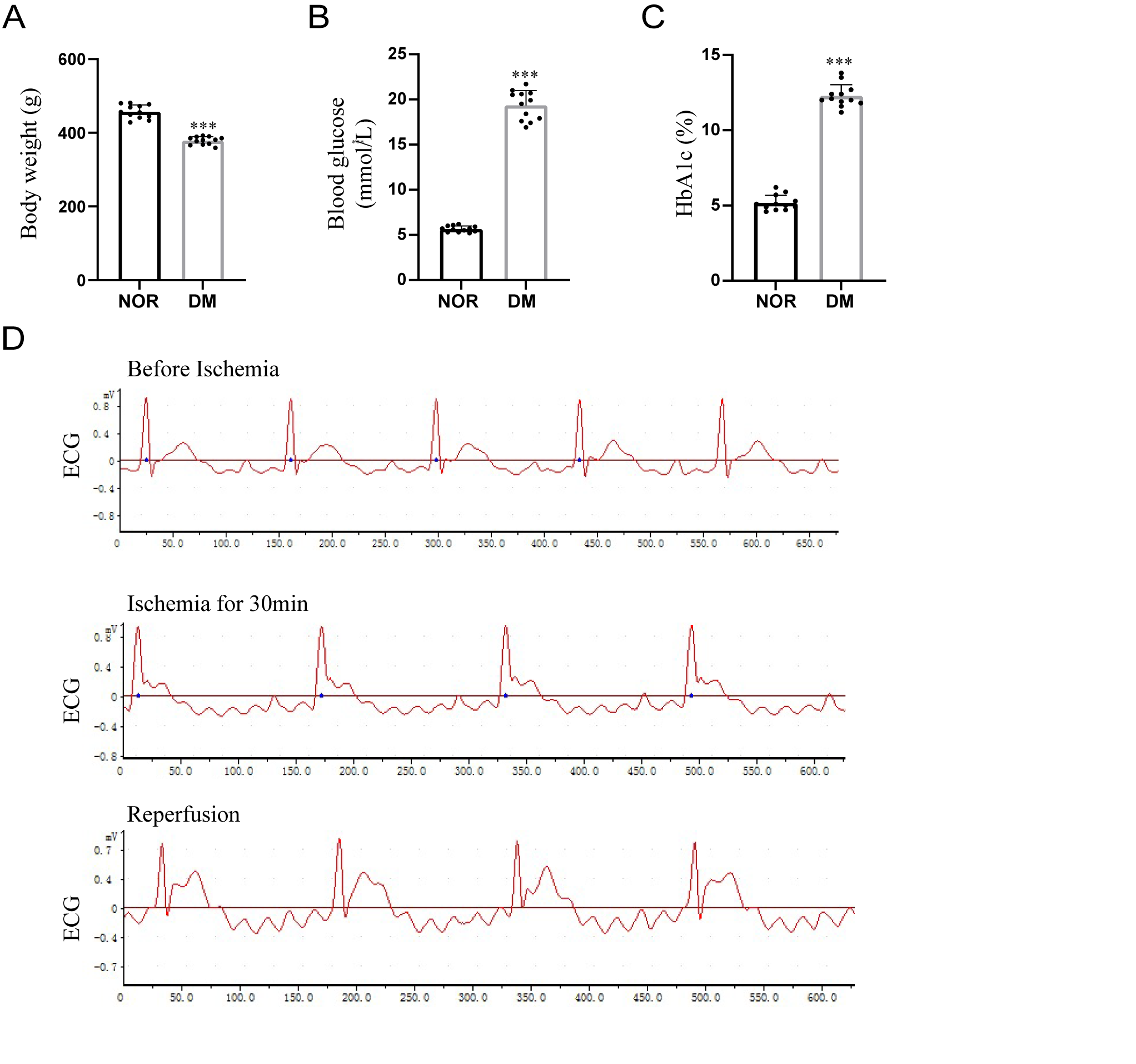

Supplement: Supplementary file 2 [file Image2.tif]

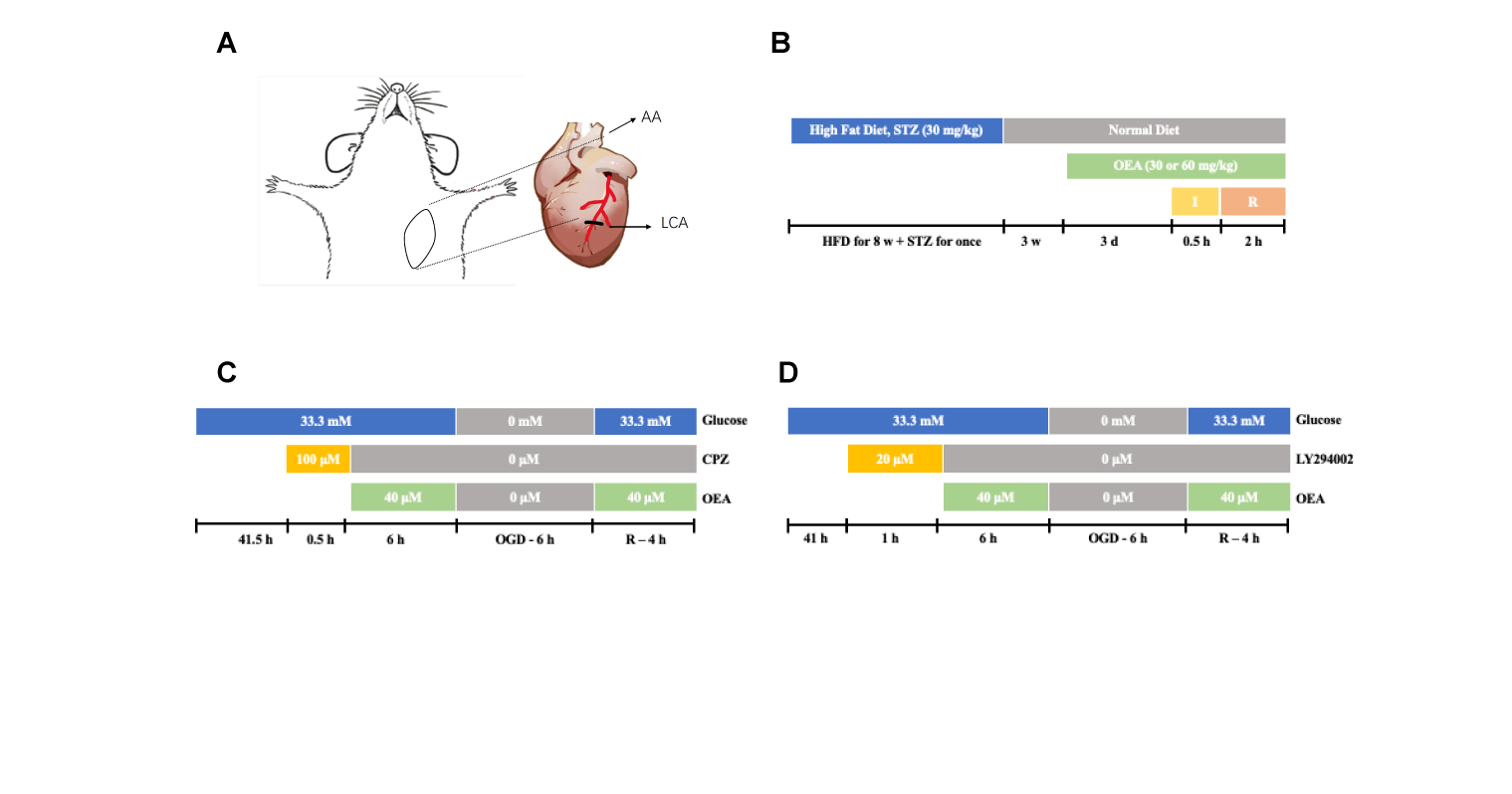

Supplement: Supplementary file 3 [file Image1.TIF]
